# Pluripotent stem cell-based immunotherapy: advances in translational research, cell differentiation, and gene modifications

**DOI:** 10.1093/lifemedi/lnaf002

**Published:** 2025-01-18

**Authors:** Qi Lei, Hongkui Deng, Shicheng Sun

**Affiliations:** Department of Cell Biology, School of Basic Medical Sciences, Peking University Stem Cell Research Center, Peking University Health Science Center, Beijing 100191, China; Department of Cell Biology, School of Basic Medical Sciences, Peking University Stem Cell Research Center, Peking University Health Science Center, Beijing 100191, China; Changping Laboratory, Beijing 102206, China; Changping Laboratory, Beijing 102206, China; Murdoch Children’s Research Institute, The Royal Children’s Hospital, Parkville, Victoria 3052, Australia

**Keywords:** immunotherapy, pluripotent stem cells, off-the-shelf, gene-editing, differentiation

## Abstract

Cell-based immunotherapy, recognized as living drugs, is revolutionizing clinical treatment to advanced cancer and shaping the landscape of biomedical research for complex diseases. The differentiation of human pluripotent stem cells (PSCs) emerges as a novel platform with the potential to generate an unlimited supply of therapeutic immune cells, especially when coupled with gene modification techniques. PSC-based immunotherapy is expected to meet the vast clinical demand for living drugs. Here, we examine recent preclinical and clinical advances in PSC-based immunotherapy, focusing on PSC gene modification strategies and differentiation methods for producing therapeutic immune cells. We also discuss opportunities in this field and challenges in cell quality and safety and stresses the need for further research and transparency to unlock the full potential of PSC immunotherapies.

## Introduction

Adoptive transfer of engineered immune cells has transformed treatment for advanced malignancies, particularly in cancer therapy. It has been 12 years since the first success of chimeric antigen receptor (CAR) T cell therapy for acute lymphoblastic leukemia, which has paved the way for cell immunotherapy [1]. Following that, the US FDA has approved multiple CAR-T cell therapies, significantly impacting cancer treatment (Table 1). CAR-T cells are being examined for treating other complicated diseases including autoimmune diseases, infectious diseases, and fibrosis diseases [21]. In 2022, the FDA approved tebentafusp-tebn (Kimmtrak®), a bispecific T cell engager protein which can help T cell recognize, attack and kill tumor cells. In 2024, the first tumor-infiltrating lymphocyte (TIL)-based therapy Amtagvi (Lifileucel®) was approved by FDA, and subsequently, afamitresgene autoleucel (Tecelra®) as the first TCR-T therapy for synovial sarcoma, marking a milestone in immunotherapy [22]. Not only for T cells, natural killer (NK) cells and myeloid cell types have been investigated for devising immunotherapy, which have greatly diversified the range of cell immunotherapies, providing treatment options for patients with challenging diseases.

**Table 1.** FDA and NMPA approved CAR T cell therapies.

Generic name/nickname	Trade/brand name	CAR structure	Target	Indications	Date of approval	Refs.
	FDA approved CAR-T cell therapies					
Tisagenlecleucel/ Tisa-cel	Kymriah®	Anti-CD19 + CD8α + 4-1BB + CD3ζ	CD19	r/r B-cell precursor ALL, r/r large B-cell lymphoma, r/r FL	30 August 2017	[2, 3]
Axicabtagene ciloleucel/Axi-cel	Yescarta®	Anti-CD19 + CD28 + CD3ζ	CD19	r/r large B-cell lymphoma, r/r FL, large B-cell lymphoma that is r/r after first-line treatment	18 October 2017	[4–6]
Brexucabtagene autoleucel/Brexu-cel	Tecartus®	Anti-CD19 + CD28 + CD3ζ	CD19	r/r MCL, r/r B-cell precursor ALL	24 July 2020	[7]
Lisocabtagene maraleucel/Liso-cel	Breyanzi®	Anti-CD19 + IgG4 + CD28 + 4-1BB + CD3ζ	CD19	r/r large B-cell lymphoma et al., r/r CLL or SLL, r/r FL, r/r MCL	5 February 2021	[8, 9]
Idecabtagene vicleucel/Ide-cel	Abecma®	Anti-BCMA + CD8α + 4-1BB + CD3ζ	BCMA	r/r multiple myeloma	26 March 2021	[10, 11]
Ciltacabtagene autoleucel/Cilta-cel	Carvykti®	Anti-BCMA + CD8α + 4-1BB + CD3ζ	BCMA	r/r multiple myeloma	28 February 2022	[12, 13]
	NMPA approved CAR-T cell therapies					
Axicabtagene ciloleucel/ Axi-cel	Yescarta®	Anti-CD19 + CD28 + CD3ζ	CD19	r/r large B-cell lymphoma, r/r large B-cell lymphoma et al., r/r FL	22 June 2021	[4–6]
Relmacabtagene Autoleuce/Relma-Cel	Carteyva®	Anti-CD19 + IgG4 + CD28 + 4-1BB + CD3ζ	CD19	r/r large B-cell lymphoma, r/r FL	1 September 2021	[14, 15]
Equecabtagene Autoleucel/equ-cel	Fucaso®	Anti-BCMA + CD28 + 4-1BB + CD3ζ	BCMA	r/r multiple myeloma	30 June 2023	[16, 17]
Inaticabtagene Autoleucel Injection	YuanRuiDa®^b^	Anti-CD19^a^ + 4-1BB + CD3-ζ	CD19	r/r B-cell precursor ALL	8 November 2023	[18]
Zevorcabtagene Autoleucel/zevor-cel	SaiKaiZe®^b^	Anti-BCMA + CD8α + 4-1BB + CD3ζ	BCMA	r/r multiple myeloma	23 February 2024	[19]

^a^A novel anti-CD19 CAR with the scFv derived from hybridoma HI19α [20].

^b^There is no English translated name.

r/r, relapsed or refractory; ALL, acute lymphoblastic leukemia; MCL, mantle cell lymphoma; FL, follicular lymphoma; CLL, chronic lymphocytic leukemia; SLL, small lymphocytic lymphoma; BCMA, B-cell maturation antigen.

Nonetheless, these treatments rely on the use of primary cells isolated from patients or healthy donors. This significantly limits their clinical application due to issues including the variability across donors, insufficient number of cells, dysfunction after long-term expansion *in vitro*, and low efficiency of gene modifications.

**Figure 1.**
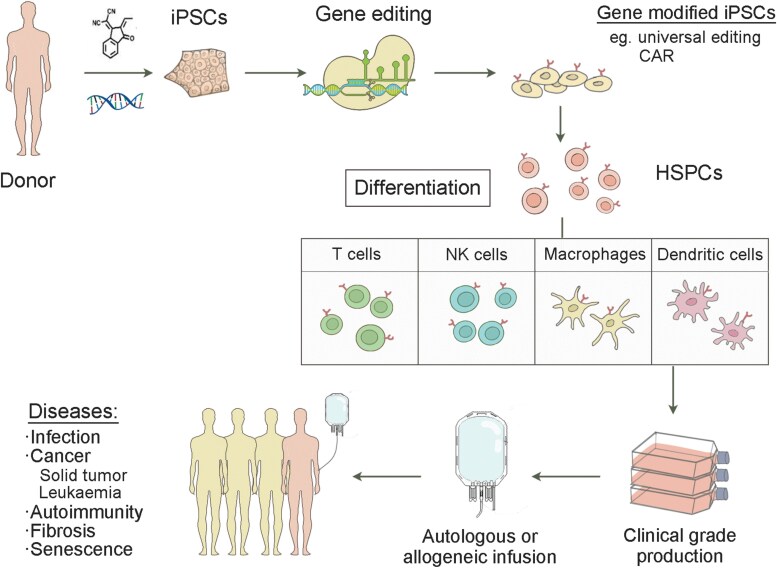
**Overview of iPSC-based immunotherapy.**This figure illustrates the process of using gene editing techniques in iPSCs to generate different immune cells for therapeutic purposes. The process begins with the gene modification of iPSCs, which can be tailored for universal editing or off-the-shelf applications, depending on the therapeutic needs. The modified iPSCs are then differentiated into hematopoietic stem and progenitor cells (HSPCs), which serve as the precursors for a range of immune cells. These cells can be further differentiated into several types of immune cells for therapeutic applications.

Pluripotent stem cells (PSCs) are promising to generalize these treatments by producing desired off-the-shelf therapeutic immune cells. PSCs are self-renewable that can unlimitedly differentiate into all cell types found within the human body [23]. Moreover, gene-editing tools optimized for PSCs have enabled functional customization of PSC-derived cells, making them highly promising for clinical applications [24]. The field of PSC-based immunotherapy has rapidly growing within both academic research and biotech industry. The differentiation of PSCs has facilitated the production of specific immune cell types, including T cells, NK cells, and myeloid cells, with several products featuring NK cells and T cells that have been tested clinically for cancer, fibrosis, and lupus (Fig. 1).

However, challenges are raised by the immature and hybrid nature of immune cells generated *in vitro*, despite that they show surface markers and defined gene regulatory networks, and rudimentary functions. Indeed, these concern treatment’s effectiveness and safety. Here, we review recent studies that offers crucial insights into fine-tuning of gene modification and differentiation approaches to improve cell quality and discusses the need to overcome challenges impeding PSC-based immunotherapy.

## The landscape of clinical PSC-based immunotherapy

Biotech industries, such as Fate Therapeutics and Century Therapeutics, have been pioneering clinical studies for induced NK (iNK) cell and induced T (iT) cell products from PSCs with preliminary results released through a non-peer-reviewed manner (Table 2). Although donor-derived allogenic reactive NK cells have been tested safe and effective in treating hematologic malignancies and melanoma [25, 26], the clinical efficacy of universal off-the-shelf iNK cells undergoing phase I trials has been suboptimal. Century Therapeutics developed an engineered CD19-CAR iNK cell therapy, CNTY-101, targeting B-cell malignancies [27], while FT500 [28], FT522, FT516 [29–31], FT536 [32, 33], FT538 [34, 35], FT576 [36, 37], and FT596 [38] are developed by Fate Therapeutics. Even with various sophisticated genetic modifications in iNK cells, clinical outcomes are not satisfactory that several pipelines suspended, excepting FT522 that is remained in phase I clinical trials by the time this paper is written.

**Table 2.** Summary of iPSC-derived immune cells in clinical trials

Products	Genetic engineering	Trial/phase	Indications	Combination	Sponsors
iPSC-NK cell					
FT500	N/A	NCT03841110/phase 1	Advanced solid tumors	With ICI therapy and IL-2	Fate Therapeutics
		NCT04106167	Multiple tumor types	N/A	
FT516	hnCD16	NCT04363346/phase 1	COVID-19	N/A	Fate Therapeutics
		NCT04023071/phase 1	Acute myeloid leukemia (AML), B-cell lymphoma	With obinutuzumab	
		NCT04630769/phase 1	Ovarian cancer	With enoblituzumab and IL-2	
		NCT04551885/phase 1	Advanced solid tumors	With avelumab	
FT538	hnCD16/CD38KO/IL-15RF	NCT04714372/phase 1	AML, myeloid leukemia, monocytic leukemia	With daratumumab	Fate Therapeutics
		NCT05069935/phase 1	Advanced solid tumors	With monoclonal antibodies	
		NCT04614636/phase 1	AML, multiple myeloma	With daratumumab or elotuzumab	
		NCT05708924/phase 1	Solid tumor	With enoblituzumab	
		NCT05700630/phase 1	Persistent low-level HIV viremia	With vorinostat	
FT596	hnCD16/anti-CD19 CAR/IL-15RF	NCT04555811/phase 1	NHL, diffuse large B cell lymphoma, high-grade B-cell lymphoma	With rituximab	Fate Therapeutics
		NCT04245722/phase 1	B-cell lymphoma, CLL	With anti-CD20 monoclonal antibodies	
FT536	hnCD16/CD38KO/anti-MICA/B CAR/IL-15RF	NCT05395052/phase 1	Advanced solid tumors	With monoclonal antibodies	Fate Therapeutics
		NCT06342986/phase 1	Gynecologic cancer, ovarian cancer, fallopian tube cancer, primary peritoneal cavity cancer	N/A	
FT576	BCMA-CAR/hnCD16/IL15RF/CD38KO	NCT05182073/phase 1	Multiple myeloma	With daratumumab	Fate Therapeutics
FT522	CD19-CAR/hnCD16/IL15RF/ADR/CD38KO	NCT05950334/phase 1	Relapsed/refractory B-cell lymphoma	With rituximab	Fate Therapeutics
CNTY-101	sIL-15/EGFRt/anti-CD19 CAR	NCT05336409/phase 1	B-cell malignancies	With IL-2	Century Therapeutics
N/A	N/A	NCT06245018	Solid tumor	N/A	Nuwacell Biotechnologies
iPSC-T cell					
FT819	TCR KO/anti-CD19 CAR	NCT04629729/phase 1	Lymphoma, CLL, precursor B-cell ALL	With IL-2	Fate Therapeutics
		NCT06308978/phase 1	Systemic lupus erythematosus	With chemotherapy	
FT825/ONO-8250	TCR KO/HER2 CAR/IL7RF/hnCD16a/CXCR2/TGFb redirect/CD38KO	NCT06241456/phase 1	Advanced solid tumors	With monoclonal antibody	Fate Therapeutics/Ono Pharmaceutical

FT576 was an iNK cell therapy targeting B-cell maturation antigen-positive conditions. It has been administered to nine patients with relapsed/refractory multiple myeloma. Within the six-patient group that received a single dose (monotherapy), only one refractory patient achieved a very good partial response, while the others remained in disease condition, including two progressive cases and three stable cases. There were other three patients who received a single dose plus daratumumab treatment; in this group, one achieved a partial response (PR), one achieved stable disease, and another achieved a minor response (MR). No dose-limiting toxicities nor events of any grade of cytokine release syndrome, immune effector cell-associated neurotoxicity syndrome, or graft-versus-host disease (GvHD), were observed [39]. The other product, FT522, has entered phase I clinical trial, but its results have not been published.

Furthermore, Fate Therapeutics is testing two CAR iT cell products, FT819 [40] and FT825. FT819 is currently undergoing clinical evaluation as a potential therapeutic approach for CD19^+^ relapsed/refractory B-cell malignancies. The first clinical trial was developed with first 15 patients. The treatment led to an objective response in four patients, comprising three complete responses and one partial response by a single dose treatment, while multiple dose group did not show obvious benefits. Notably, two patients with Richter transformation showed disease progression or stable disease condition suggested by positron emission tomography examination. No detection of anti-product human leukocyte antigen (HLA) Class I antibodies in patients’ serum. These findings suggest that the therapy holds promise for certain patients, but the efficacy varies among different disease conditions.

FT825 is a new pipeline under development for treating solid tumors against HER2, which incorporates seven genetic manipulations. These include T cell receptor (TCR) knockout and HER2-CAR transduction, and the expression of a novel high-affinity 158V, non-cleavable CD16 Fc receptor to enhance antibody-dependent cellular cytotoxicity (ADCC), CD38 knockout to promote persistence and function in high oxidative stress environments, overexpression of a synthetic IL-7/IL-7 receptor fusion (IL-7RF) and a CXCR2 receptor to respectively promote T-cell stemness and cell trafficking, and finally, it includes a synthetic TGFβ receptor to redirect immunosuppressive signals in the tumor microenvironment. Despite this advancement, it has been only tested on a preclinical setup and the clinical trial was claimed to be launched in January, 2024.

Collectively, phase I trials for iNK cell and iT cell therapies showed mixed results according to partially disclosed knowledge; FT819 and FT576 show potential in treating B-cell malignancies, while advanced genetic modifications in FT825 for solid tumors are promising but still preclinical.

## Advances in preclinical investigation

### T cells

**Figure 2.**
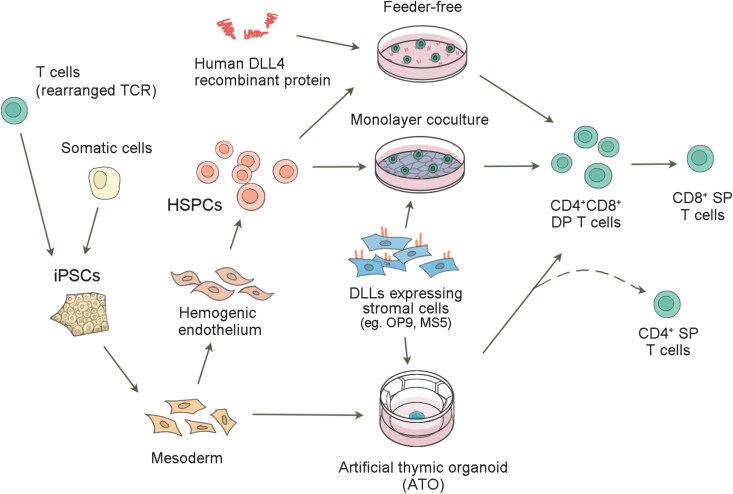
**Schematic illustration of feeder-free human T cell induction and differentiation process.**The process involves the use of recombinant proteins, such as human DLL4, to induce the differentiation of HSPCs into various T cell lineages. The depicted coculture systems utilize DLL-expressing stroma cells (e.g. OP9, MS5), which mimic the thymic microenvironmental NOTCH signals. These cells support the development of T cells through different stages. iPSCs reprogrammed from T cells whose TCR locus are rearranged are being used as a preferable option for differentiating T cells. DP, double positive; SP, single positive.

Recent preclinical efforts have yielded more promising results, demonstrating competitive efficacy in animal models. Importantly, methods of these studies are disclosed, providing insights into the refining of relevant technologies. Several independent groups have used feeder-free differentiation systems to induce T cells from PSCs, which some showed therapeutic effects [41, 42] (Fig. 2). Jing et al. generated EZH1-repressed CAR-iT cells, which upon injection exhibited comparable anti-tumor efficacy to peripheral blood-derived CAR-T cell treatment throughout the 7-week duration of the experiment [41]. Shoichi Iriguchi et al. demonstrated that their CAR-iT cells exhibit long-term persistence following tumor clearance, leading to a significant improvement in overall survival for tumor-bearing mice up to 80 days post-injection [42]. These systems can be feasibly applied in clinical production, while the robustness and reproducibility have been problematic due to the intrinsic limitation of differentiation systems.

On the other hand, stromal cell-based differentiation systems are more prevalent that are stably used for T cell differentiation. MS5 and OP9 are two mouse stromal cell lines to carry notch ligand expression, which support T cell differentiation [43, 44]. Originally, OP9 monolayer cultures were developed and commonly used (Fig. 2). In 2013, Themeli et al. utilized CD19 CAR-T-PSCs (T cell-derived PSCs expressing CD19 CAR construct) to generate cytotoxic lymphocyte, initiating tumor regression in Raji Burkitt lymphoma mouse model [45]. Since then, there have been studies optimized this OP9-based system and observed iT cell-inducing tumor remission in tumor-bearing mice [46–48]. In a further developed inducing system, Van der Stegen et al. efficiently induced CAR-T-PSCs into functional CD8αβ-positive CAR T cells, demonstrating complete and durable responses in NALM6-injected mice. Compared with conventional CD8 CAR-T cells, their iT cells exhibited equivalent therapeutic efficacy [49]. In addition, using MS5 stromal cells engineered with notch ligand, Crooks and colleagues established a 3D organoid system, known as the artificial thymic organoid (ATO) [44] (Fig. 2). The iT cells carrying a CD19 CAR, derived from ATOs, showed therapeutic efficacy in an aggressive mouse tumor model, significantly enhancing mouse survival for up to 60 days compared to the untreated group that died from day 30 [50]. These compelling preclinical outcomes provide early-stage evidence that supports the promise of iT cells for therapy.

T cells with their ability to form memory cells and the enhanced antigen recognition provided by CAR construct, have shown impressive responses against various malignancies. However, challenges such as GvHD, cytokine storm, and tumor antigen escape have prompted the exploration of new therapies. In contrast, NK cells, part of the innate immune system with a broader target range and activation through MHCI downregulation [51], offer a safer alternative, particularly in allogeneic grafts, due to their lower risk of GvHD [52].

### NK cells

Based on well-established “spin-embryoid body” technique [53], Kaufman and colleagues achieved a notable success in producing clinical-grade NK cells that have been used to develop anti-tumor treatments [54]. Aside from differentiation system optimization, modification strategies were further utilized to enhance the persistence and killing capacity of NK cells. Cytokine-inducible SH2 domain-containing protein (CISH), a member of the suppressor of cytokine signaling protein family, is potent to inhibit cytokine-downstream JAK–STAT signaling by negative feedback mechanisms [55]. Biallelic *CISH*-knockout iNK cells showed enhanced sensitivity of IL-15 signaling, persisting longer in the peripheral blood of recipient than non-edited cells or peripheral blood-derived NK cells, which contributed to 60% of the mice showing complete tumor clearance and long-term survival over 100 days [56]. Introduction of reconstructive IgG Fc receptors CD16a or/and CD64, the key mediators of ADCC for NK cells [57], also improved the efficacy of PSC-based antitumor efficacy [58, 59]. Snyder et al. developed a recombinant FcγR (CD64/16A) by substituting the extracellular domain of CD16a with CD64, which has higher affinity to mediate stronger ADCC in conjunction with trastuzumab [59, 60]. In line with it, introduction of CD16a together with IL-15 and knockout of CD38 generated a virus-induced phenotype in NK cells, which addressed the limitations including relatively lifespan and limited expansion capacity of NK cells shown in traditional NK cell-based immunotherapies [58]. The synergistic effect of these induced NK cells and a monoclonal antibody against CD38, daratumumab, has achieved to a 99% tumor reduction. These findings underscore the potential of gene-modified PSC-derived NK cells as a powerful tool in combined therapy against cancer.

### Myeloid cells

Myeloid cells are attractive in immunotherapy for cancer treatment, as they naturally infiltrate tumors and can be targeted to improve the immunosuppressive tumor microenvironment (TME). Gill and colleagues demonstrated the proof-of-concept, in which a second-generation CARs containing a CD3ζ domain induced M1-like macrophages (iMacs), and a single infusion of CAR-Macs in mice bearing xenograft solid tumor improved significantly overall survival rate from 60 days up to 100 days [61]. For PSCs, Shah et al. introduced a prostate stem cell antigen-targeting CAR with co-engineered mIL-15 and a suicide switch in the form of a truncated version of epidermal growth factor receptor (tEGFR) into PSCs [62]. These CAR-iMacs reduced pancreatic tumor burden in mice and improved survival from 40 to 60 days compared to both saline and mock treatment. Lei et al. engineered toll-like receptor 4 intracellular toll/IL-1R (TIR) domain-containing CARs, which conferred iMacs with both target engulfment capability and antigen-dependent M1 polarization, as well as the ability to modulate the tumor microenvironment [63]. Their CAR-iMacs exhibited significant antitumor activity, extending the overall survival from 60 to 100 days in HepG2 cell xenograft models. Shen et al. repolarized CAR-iMacs *in vivo* by continuous administration of IFN-γ and monophosphoryl lipid A to activate innate immune responses [64]. Their activated CAR-iMacs acquired a tumoricidal phenotype, combined with T cell treatment, leading to a prolonged survival rate of over 100 days in human ovarian cancer models compared to the initial 70 days injected with unstimulated CAR-iMacs. Besides CAR downstream signaling regulation and cytokine activation, genetic modulating the metabolic gene which controls cellular metabolism and pro-inflammatory activity of macrophages may also be a potential method to induce M1 state; *ACOD1*-depleted CAR-iMacs combined with immune checkpoint inhibitors (ICIs) enhanced their tumor suppressing effect [65].

Cell therapies that use PSC-derived dendritic cell (iDCs) focus on their capacity for antigen presentation and chemotaxis—as a type of cancer vaccine—to modulate the tumor microenvironment and attract cytotoxic lymphocytes [66]. In addition, the combination of iDCs with ICI and radiotherapy may achieve potent synergistic anti-tumor effects better than single-agent treatments, characterized by increased infiltration of CD8 T cells or NK cells into solid tumors, enhancing the overall immune response against cancer [67, 68]. The introduction of c-Myc into myeloid progenitor cells, enabling indefinite proliferation and differentiation into antigen-presenting DCs upon IL-4 and GM-CSF stimulation [69]. Subsequently, these c-Myc-transfected PSC-derived myeloid cells artificially expressing IFN-α positively regulate donor’s XCR1^+^ DCs and CD8^+^ T cell activation, enhancing anti-PD-L1 therapy’s efficacy against ICI resistance [67]. Furthermore, these myeloid cells were overexpressed with GM-CSF, expressing certain surface markers that resembled bone marrow DCs. When combined with ICIs, they showed improved anti-tumor activity [70].

Granulocytes are attractive in tumor immunotherapy due to their ability to infiltrate solid tumors and regulate immunosuppressive microenvironment. Bao and colleagues genetically expressed an anti-glioblastoma CAR in PSCs and generate functional induced CAR-neutrophils carrying tumor microenvironment-responsive nanodrugs [71, 72]. CAR PSC-neutrophils can travel across blood-brain barrier and alleviate tumor burden in a glioblastoma model; but the overall survival rates of animals showed limited advantages compared to the control groups. In addition, eosinophils were efficiently generated from PSCs, and eosinophils showed some antitumor effects in solid tumors when combined with CAR-T cell therapy [73].

In summary, while phase I clinical trials of PSC-based immunotherapy have shown mixed outcomes, certain preclinical studies using advanced genetic modifications offer promising results. Myeloid cells, including CAR-iMacs and iDCs, are emerging as promising therapeutic agents due to their inherent tumor-infiltrating properties and their ability to modulate the tumor microenvironment. However, it continues to confront challenges in achieving consistent and reliable outcomes, largely attributable to the current limitations of differentiation methodologies.

## Differentiation of immune cells from PSCs

**Figure 3.**
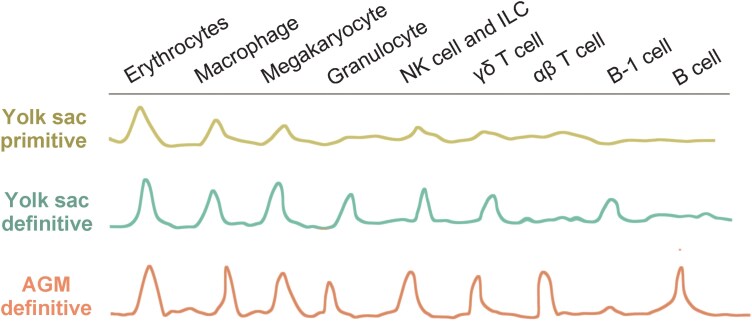
**Schematic representation of immune cell lineages associated with distinct waves of embryonic hematopoiesis.**The figure also includes references to different sites of hematopoiesis during embryonic development. The YS initiates the formation of blood cells, in which the first phase (primitive) produces red blood and myeloid cells are produced, and the second phase (definitive) generates certain myeloid and lymphoid immune cell types. The AGM region is another important site for definitive hematopoiesis where more mature and diverse immune cells are generated.

PSC differentiation to immune cells mirrors the natural embryonic hematopoietic development that initially forms blood progenitors, followed by commitment into different immune lineages. Accurately recapitulating this process is complex due to the layered hematopoiesis within mammalian embryos, with each wave leading to distinct immune cell outcomes, which is reviewed elsewhere [74–76]. Briefly, as intensively studied in the mouse, the first wave occurs at embryonic day 7 (E7) in the yolk sac (YS), generating cells that develop into erythroid, macrophage, and megakaryocytic lineages. The second wave, from E8.5 to E9, produces erythroid cells, myeloid cells, fetal B cells and γδ T cells. Most crucially, the definitive wave of hematopoiesis at E10.5 to E11.5 in the aorta-gonad-mesonephros (AGM) region generates multipotent cells with a strong capacity for lymphoid differentiation (Fig. 3). T and NK cells, critical for cytotoxic and tumor killing functions, are a focus of research aiming to efficiently direct PSC differentiation toward the definitive waves for therapeutic applications.

### Lymphoid induction in vitro

Pioneering studies by the Keller laboratory proposed T cell differentiation as a surrogate readout when screening conditions for definitive hematopoiesis [77, 78]. Importantly, inhibition of TGFβ together WNT activation pathway augments the specification of definitive hematopoietic progenitors with multi-lineage potential [78–82]. Conversely, Atkins et al. identified that activation of Activin/Nodal pathway during mesoderm formation recapitulated YS-like hematopoiesis, in which blood progenitors expressed CD235a without the potential to differentiate into conventional αβ T cells but γδ T cells [83]. The synergistic effect of the combination of WNT and Activin/Nodal signaling benefits the induction of lymphoid cells [84]. In addition, knocking down the epigentic regulator, polycomb group protein *EZH1*, resulted in the generation of HSPCs that were more akin to those produced by embryonic definitive hematopoiesis with multi-lymphoid potential [41, 85].

The endothelial-to-hematopoietic transition (EHT) is also critical to the cell outcomes [86]. This transition influences the characteristics of hematopoietic progenitors that emerged from a subset of arterial endothelial cells (AECs) marked by the expression of the NOTCH ligand Delta-like ligand 4 (DLL4) that supports lymphoid differentiation [79, 87]. Inhibiting NOTCH signaling by the small molecule DAPT profoundly diminished hematopoiesis [88], while stimulating with recombinant DLL4 protein facilitated blood generation. When cultured on a DLL4-coated plate, sorted CD34^+^ endothelial cells exhibited an approximately 80-fold increase in T progenitor cell output, suggesting that manipulating EHT altered the lineage potential of HSPCs [89]. The DLL4 naturally expressed by hemogenic AECs can also support the arising of lymphoid committed RAG1^+^ progenitor cells, which subsequently differentiated into CD4^+^CD8^+^ T cells and innate lymphoid cells (ILCs), including NK cells, without the need for exogenous DLL4 employment [79, 81].

### T cells

T cell differentiation from PSCs follows essential process of thymopoiesis, where the NOTCH signaling plays a pivotal role. Thymic epithelial cells express NOTCH ligands, such as DLL4 and DLL1, which have distinct interaction strength and partially overlap functions; either DLL4 or DLL1, or both, can support T cell differentiation *in vitro*. While compared to DLL1, DLL4 is more effective, particularly expressed at a lower level by stromal cells [90]. In the ATOs [91], MS5-DLL4 produced a higher purity of hematopoietic and T cells with a greater output of mature CD8 single-positive (SP) T cells compared with those with DLL1. Parallel comparison of human NOTCH ligands DLL1, DLL4, Jagged-1 (JAG1), and JAG2 revealed that only DLL4 or JAG2 can support double-positive (DP) cell differentiation, with DLL4 being significantly more efficient than JAG2 [49]. As such, DLL4 is a preferred option for inducing T cell from PSCs.

DLL4 recombinant proteins in an immobilized form provide a feeder cell-free solution for clinical translation. It often incorporates additional recombinant proteins (such as vascular cell adhesion molecule 1 (VCAM1) and retronectin) in the form of plate coating or bead binding [42, 84, 92, 93]. VCAM1 and retronectin, as integrin ligands, are crucial for guiding thymocyte migration and adhesion [94, 95]. They also function in synergy to enhance the NOTCH signaling activity [89, 92]. CXCL12, a thymic chemokine, supports the proliferation and development of thymocytes in conjunction with DLL4 [96, 97]. The CXCR4/CXCL12 axis is known to act as a co-stimulatory pathway during β-selection, influencing the localization of immature thymocytes, survival signals, and proliferation [98]. Furthermore, CXCL12 (SDF1a) combining a selective p38 MAPK inhibitor, SB203580, significantly boosted the generation of DP T cells by approximately 5000-fold, and upregulated the expression of key transcription factors essential for T linage commitment, such as BCL11B, TCF7, and GATA3 [42]. SB203580 was considered to promote the survival of immature hematopoietic progenitor cells, while its molecular mechanism was undefined. A cytokine screen conducted by Edgar et al. revealed that IL-3 and TNFα significantly enhance the yield of CD5^+^CD7^+^ T cell progenitors, with IL-3 promoting the proliferation of myeloid-biased CD34^+^ HSPCs and TNFα inducing T-lineage differentiation by modulating NOTCH target genes, leading to a substantial expansion of early T cell progenitors [93].

Interestingly, the differentiation outcomes vary between the feeder-free system and the ATO system. It is speculated that the 3D structure of ATO promotes valence and/or duration of contact between T cell precursors and selective ligand, allowing T cell maturation from the DP to the SP stage, which heavily rely on TCR–MHC interaction that is hardly supported in monolayer cell culture. The ATO system has been also useful for elucidating the mechanism of hematological diseases, providing clues for therapeutic treatments [99, 100]. Nevertheless, these feeder cell-based systems are limited in clinical transition due to its reliance on animal-derived feeder cell line and serum. In the feeder-free system, the transition from the DP to SP requires a stimulation by anti-CD3 antibody due to the lack of peptide-major histocompatibility complex (pMHC). Interestingly, CD3 stimulation only generates CD8^+^ T cells but not CD4^+^ T cells. Similarly, the ATO system generates predominantly CD8^+^ T cells and rarely produces CD4^+^ T cells only in prolonged culture extending to 5–7 weeks [91, 101].

### NK cells

Initially, PSC-derived hematopoietic progenitors were able to differentiate to NK cells when cocultured with mouse feeder cell line with added cytokine cocktails (IL-15, IL-7, IL-3, SCF, Flt3L) [102], which is now replaced by the stromal cells spontaneously induced in the embryoid bodies (EBs) during PSC differentiation. The stromal cells generated in the EBs expressed the endothelial markers CD31 and CD73, and more importantly, they expressed MHCI molecules (HLA-A, B, C, and HLA-E) that are known crucial for NK cell maturation [54]. Immature NK cells express inhibitory killer cell immunoglobulin-like receptors (KIRs) and LY49 receptors, with these receptors binding to homologous MHCI molecules in the periphery to mature into functional NK cells [103]. iNK cells can function similarly to allogeneic PBMC derived-NK cells, by the expression of lineage associated markers such as CD56, CD16, CD94, as well as NKp44, NKp46, NKG2A, NKG2D. Notably, these iNK cells express a lower expression level of NKG2C. They secrete anti-tumor cytokines (IFN-γ and TNF-α), and cytotoxic molecules such as perforin and granzyme-B, effectively killing HLA-null tumor cell lines like K562 cells [54, 104].

NK cell ontogeny has also complicated PSC differentiation as they originate from different sources during embryonic hematopoiesis [105]. Using PSCs mimicking early human AGM hematopoiesis and lymphopoiesis, Sun et al. found that a group of RAG1^+^ lymphoid precursors arising directly from AECs, which can develop into NK and ILC lineages through the involvement of NOTCH and IL-7 pathway, with the fate decision between T and innate lymphoid lineage being determined by the level of the IL-7 [79]. Besides the classical developmental path from HSCs to lymphoid progenitors, NK cells can also differentiate from human peripheral blood-derived myeloid progenitors [106], suggesting alternative trajectory exists during embryonic development for the generation of NK cells. Dege et al. isolated a group of EMPs (Kit^+^CD16/32^+^CD41^+^) from E9.5 YS of murine embryo and cultured them on OP9 cells with IL-2, IL-7, and IL-15, differentiating them into NK cells [107]. Parallel studies using PSCs revealed that iNK cells derived from YS and AGM region might differ functionally, mainly in cytokine secretion and degranulation [107]. But, due to a lack of substantial *in vivo* experiments, it remains to be further explored for which NK cell source might offer more potent anti-tumor function.

### Myeloid cells

Macrophages are innate immune cells, responsible for pathogen clearance through phagocytosis and induce adaptive immune responses by presenting antigens to T cells. PSC differentiation protocols for Macs have been well established and compared previously [108]. The differentiation process does not require stringent guidance of hematopoietic commitment as that both extra-embryonic and intra-embryonic hematopoietic progenitors can differentiate into Macs. Following the induction of mesodermal progenitors, the addition of combination of cytokines, such as VEGFA, SCF, FGF2, FLT3L, TPO, IL-3, M-CSF, and GM-CSF, is sufficient to induce Macs, and using only M-CSF as the sole hematopoietic cytokine for Macs induction [109]. iMacs express markers including CD11b, CD14, CD68, CD169, and CD206 with corresponding phagocytic functions [110] and polarize to M2 or M1 phenotype under the stimulation of IL-4 or IFN, respectively [111, 112]. The yield discrepancy between different protocols remains poorly understood, but large-scale clinical-grade production can be established in bioreactor [113] or spinner [111]. The bioreactor was reported to achieve a production of approximately 1 × 10^7^–3 × 10^7^ iMac per week for more than 5 weeks, while the spinner was capable of generating a series of 18–25 harvests with single harvest yields reaching up to 6 × 10^8^ cells, which benefits clinical translations.

Dendritic cells originate from different progenitors, such as common lymphoid progenitors, common myeloid progenitors, and granulocyte-monocyte-DC progenitors, complicating PSC differentiation [114]. The process does not strictly adhere to the traditional separation of myeloid and lymphoid lineages. Although iDC studies have traced the myeloid progenitor pathway [69], the precise *in vivo* development of DC subtypes remains unclear. Studies on iDC have followed the myeloid progenitor differentiation pathway [69] despite the *in vivo* developmental trajectory for various subtypes have not been thoroughly investigated. Sontag and colleagues differentiated conventional DCs (cDC1s) and plasmacytoid DCs (pDCs) by combining SCF, FLT3L, and GM-CSF, with IL-4 inducing both cDC1 and cDC2 [115]. To generate tolerogenic DCs, cells were treated with anti-inflammatory agents like rapamycin and dexamethasone after 21 days of differentiation, which modulated moDCs before maturation with factors like IFN, TNFα, and IL-1β for 48 h [116]. These DCs, expressing CD141 and CD11c, maintain homeostasis and induce local antigen tolerance. Type 1 cDC1s are adept at generating anti-tumor CD8^+^ T cell responses through cross-presentation of exogenous antigens on MHC-I. Makino et al. generated cDC1s with Notch signaling activation via OP9-DL1 stromal cell coculture, yielding a subset with a gene profile akin to blood cDC1s [68]. However, due to the incomplete exploration of human DC subtype ontogeny, *in vitro* differentiation of “true” DCs is an ongoing challenge.

## Gene modification strategies

Gene modification techniques are instrumental in advancing cell immunotherapies by equipping PSCs with tumor-killing abilities and/or reducing the immunogenicity of PSC-derived cells. Here, we highlight two critical aspects of modification strategies with respect to the development of the T cells and the generation of off-the-shelf immunotherapies.

### Balancing CAR and TCR signals

TCR and CAR signals are functionally similar, which raises the concern that PSCs carrying a CAR (CAR-PSCs) might develop unusually by interfering with the endogenous TCR machinery during T cell differentiation. Upon activation, CAR’s intracellular domain including CD3ζ with the immunoreceptor tyrosine-based activation motif (ITAM) sets off a chain reaction similar to that activated through TCR. As such, developing T cells respond as if their TCR or CAR has been expressed, even prior to the endogenous TCR rearrangement and expose stage, causing abnormal functional development. Thus, the existence of CAR on developing T cells could oppose pre-TCR/TCR signaling activity.

Gene modifications are used to overcome problems associated with prematurely CAR expression. When CAR was knocked into the T-cell receptor α constant (TRAC) locus, it was expressed at the DP stage as controlled by the activity of TRAC’s enhancer and promoter [49]. As such, the controlled expression of CAR does not interfere the formation of pre-TCR or β-selection at the double-negative (DN) stage, facilitating the generation of CD7^+^CD5^+^ T cell precursors. Nevertheless, they also found that stronger costimulatory signal mediated by CD28 on the 1928z CAR induced an ILC-like phenotype, such as the expression of CD56 and CD8αα. To this end, a 1928z-1xx CAR, containing the two distal ITAMs that were loss-of-function mutated, alleviated the strength of the CAR signals [117], which effectively generated CD4^+^CD8^+^ DP cells. Similarly, using antigen-expressing stromal cells to support CAR-PSC differentiation generated ILC-like cells by premature activation of CAR tonic signaling that halts T cell development [118]. Replacement of the co-stimulatory domain in CD28 with 4-1BB, a weaker signal domain, facilitated an appropriate T cell development trajectory, and the resultant CAR-T cells showed normal tumor killing functions. Furthermore, PSCs transduced with the 1G4 TCR and co-cultured with MS5-DLL4 cells expressing its cognate (A2/ESO) pMHC arrested T cell differentiation at the DN stage. Instead, it produced predominantly CD8αα innate lymphocytes, which suggests that premature activation of TCR signaling induced an ILC phenotype, mirroring that found in constant expression of CARs [118].

Instead of CAR modification, manipulation of the endogenous TCR-forming programs can be also useful for the generation of functional T cells from PSCs. The Kaneko group generated PSCs from an antigen-specific T cells, so-called T-PSCs, which contained a pre-rearranged TCR gene in the genome leading to the determination of CD8 lineage without the need for rearrangement of the TCR loci [119]. Based on the exogenous transduced TCR gene or using T-PSCs, knocking out the *RAG* genes prevented the initiation of rearrangement activity and generation of unspecific TCRs [47, 101]. In this context, artificial introduction of a TCR may facilitate the differentiation as that forced TCR expression could assist the externalization of the CD3-TCR complex. An exogenously transduced WT1 TCR in PSCs led to a higher proportion of CD5^+^CD7^+^ T precursor cells [42]. Chang and colleagues introduced a specific TCR into PSCs while knocking out *RAG1* and *RAG2*, and upon transferring the cognate MHCI molecule into MS5-DLL4. They found that the outcome of TCR-iT cells exhibited better tumor control than T cells with an intact endogenous TCR [101]. Subsequently, deletion of the recombinase gene *RAG2*, prevented additional rearrangement during differentiation and reducing the risk of off-target activation and loss of antigen specificity [47].

There are studies focusing on the TCRs of innate T cells including nature killer T (NKT) cells, mucosal-associated invariant T cells, and γδT cells. These kinds of innate T cells have been reprogrammed into T-PSCs which carry the originally rearranged TCR [120–122], or directly transferred the innate TCRs into PSCs or HSPCs [123, 124]. Similar to the T cell differentiation strategies mentioned above, the hematopoietic progenitors transduced with NKT’s invariant TCRs were subsequently differentiated into cytotoxic lymphocytes, demonstrating indeed anti-tumor capabilities, reducing the risk of GvHD compared to αβ iT, thus offering higher safety [123–125].

### Making “off-the shelf” cell products

**Figure 4.**
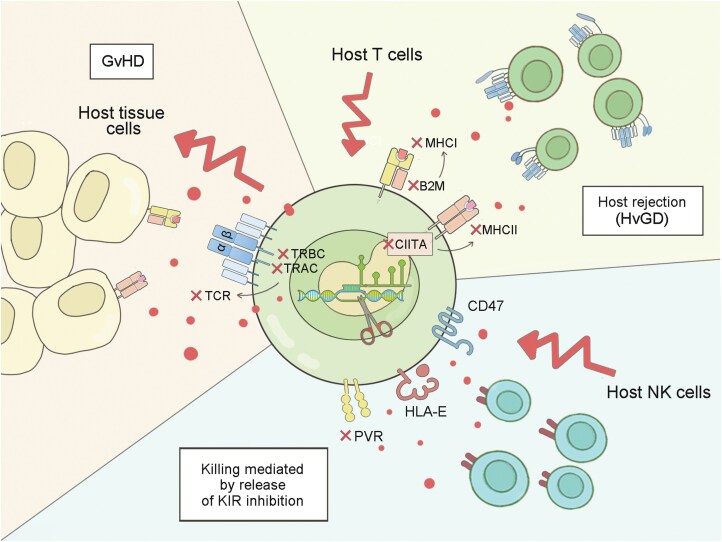
**“Universal” gene editing strategies to prevent host T cell rejection and GvHD in transplantation.**This illustration describes the complex immune interactions that occur during transplantation of iPSC-derive immune cells, focusing on the role of host T cells and the potential for both host rejection (HvGD) and graft-versus-host disease (GvHD).

Genetic modifications have been used to reduce the immunogenicity of PSC-derived immune cells to avoid GvHD (Fig. 4). Donor T cells can recognize antigens presented by MHC molecules on recipient tissue cell surfaces, triggering immunological rejection and leading to alloreaction. Moreover, even with MHC-matched donors, such as siblings, patients can develop acute and chronic GvHD in 25%–40% and 40%–60% of cases, respectively [126]. Given the risk of GvHD associated with TCR diversity, allogeneic T cells should be engineered to remove the expression of endogenous TCRs by knocking out exons of *TRAC* and/or TCRβ constant 1 (*TRBC1*) or 2 (*TRBC2*) loci [127].

Another significant obstacle is the host-mediated rejection of HLA-mismatched allogeneic donor cells, known as HvGD. To address this issue, several studies have explored the disruption of endogenous *B2M*, a HLA I subunit, to eliminate HLA I in a double-knockout scenario [128]. Furthermore, the HLA II transactivator, *CIITA*, could be also disrupted, resulting in the generation of triple-knockout T cells [129, 130] (Fig. 4). The HLA-knockout CAR-T cells have been observed to exhibit diminished allogeneic reactivity, evading host attacks and demonstrating enhanced persistence and survival capabilities [128–130]. However, the knockout of HLA I leads to the release of inhibition by MHCI-specific inhibitory receptors, such as KIRs, NKG2A, thereby activating NK cells and resulting in the rapid elimination of universal CAR-T cells by NK cells within a short span *in vitro* [129]. Overexpression of HLA-E [131] or CD47 [132] to attenuate NK cell activation can effectively mitigate the NK response against HLA-deficient universal cells. HLA-E transduction has been proven to effectively inhibit NK clearance of HLA-lacking CAR-T cells [130] (Fig. 4).

For PSC-based immunotherapy, the aforementioned modification strategies are also employed in the universal genome editing of PSC [133]. Considering GvHD could potentially occur when the differentiated iT cells express endogenous arranged diverse TCR repertoire, there are studies developed an universal iT population expressing a single clone of TCR instead of simply knocking out *TRAC* locus. To evade host immunological surveillance, Wang et al. ablated MHC-I and II in PSCs, while concurrently expressing HLA-E and knocking out the poliovirus receptor (PVR), an NK cell ligand that interacts with DNAM-1^+^ NK cells, and subsequently prevented T cell death from NK cell killing [134] (Fig. 4). This approach successfully generated hypoimmunogenic PSC-derived immune cells without disturbing the differentiation process.

Taken the above, genetic modifications of PSC-derived immune cells aim to mitigate GvHD by reducing their immunogenicity, with engineered allogeneic T cells designed to eliminate endogenous TCR expression, thus decreasing the risk of alloreactions even in MHC-matched cases. To combat HvGD, strategies include the disruption of HLA I and II, leading to the creation of triple-knockout T cells with reduced alloreactivity and enhanced survival, alongside measures to counteract NK cell activation. PSC-based immunotherapy further employs these strategies, with advancements such as the development of a universal iT population expressing a single TCR clone and the ablation of MHC-I and II in PSCs while expressing HLA-E and knocking out PVR to evade NK cell-mediated killing, generating hypoimmunogenic immune cells without disrupting differentiation.

## Opportunities and challenges

Although challenges in safety and efficacy persist influencing the durability and therapeutic efficacy of the cells, ongoing optimizations in cell differentiation and gene editing strategies are set to establish a promising treatment option. Addressing the remaining hurdles is important for the full realization of the therapeutic potential of PSC-derived immune cells.

For T cells, a critical challenge is the generation of durable memory-like T cells that can persist in long-term *in vivo*. Overexpression of the transcription factor FOXO1 has been found to enhance the stemness and metabolic fitness of CAR-T cells [135, 136], which could be transformed into iT cell differentiation. In addition, repression of EZH1 can induce a memory-like state in iT cells that can persist in the peripheral blood after transplantation, similar to conventional memory T cells [41]. Furthermore, there are innate lymphoid-like cells emerging during T cell differentiation, which show ILC (including NK cell) phenotype [137]. They typically arise post-stimulation and expansion or driven by CAR expression, expressing classical T cell markers such as CD3 and TCR while also expressing NK markers like NKp44, NKG2D, and CD56 [48, 50]. Yohei et al. also observed a decrease in CD5 expression and the emergence of CD8αα. They eliminated these innate-like cells by screening cell line and sorting NKp44^−^ population [48], whereas CD56 is considered an adhesion molecule that could be potentially expressed by conventional T cells [50]. Understanding the differentiation and divergence of NK cells and ILCs from adaptive lymphocyte fates is essential for optimizing T cell therapies.

In addition, CD4^+^ T cells are a subset of T cells that play a crucial role in the adaptive immune response, which was demonstrated in recent CAR-T clinical outcomes. The CD4 populations exhibit highly activated cytotoxicity in patients with sustained clinical remission, dominating the CAR T cell population at later time points [138]. And, the level of IFN-γ secretion by CD19-CAR CD4^+^ T cells positively correlates with patients’ survival rates [139]. However, *in vitro* differentiation of functionally mature CD4^+^ SP T cells has posed significant challenges due to the limited understanding of the underlying factors governing CD4/CD8 lineage commitment. The MHC-class-II may play a critical role for CD4 SP cells development during positive selection [140]. Yano et al. screened an iPS cell line which efficiently generated induced CD4^+^ (iCD4^+^) SP cells in ATO and utilized a cocktail to drive the expression of *FOXP3*, a master transcription factor of regulatory T cells (Treg). These HLA-A2 CAR-transduced iCD4^+^ Treg-like cells suppressed GvHD in NSG mice treated with A2^+^ human PBMCs, as that natural CD25^+^CD127^−^ Treg cells did [141]. However, the ATO system involving murine-derived stromal cells may present a barrier to clinical translation. Future efforts may focus on developing feeder-free and chemically defined systems to create clinically applicable systems.

On the other hand, the clinical trials evaluating the efficacy of iNK cells have generated mixed results limited by the suboptimal longevity and effectiveness. iNK cells also showed reduced activity and functionality compared to NK cells derived from human donors. While most studies demonstrated the capacity for tumor destruction, they also highlight the requirement for a large number of NK cells to be administered; the presence of non-engineered NK cells in the peripheral blood is typically detectable for 2 weeks following administration [142, 143]. Given that conventional NK cells have a natural lifespan of approximately two weeks in humans [144], the inability of NK cells to form long-lasting memory cells, like T cells, poses a significant challenge. Even with the use of IL-15 or IL-2 to induce memory-like persistent characteristics in NK cells, they hardly respond to immune stimuli, proliferate, and maintain functionality over the long term after multiple rounds of activation like T cells [145]. Thus, this limitation is intrinsic to the nature of NK cells themselves, which may need an intensive level of genetic engineering toward an optimal efficacy.

In summary, PSC-based therapy is transforming biomedical research, potentially offering a powerful platform to produce genetically engineered “off-the-shelf” cells for clinical treatment. Besides T and NK cells playing dominant roles in eliminating target cells, myeloid cells will be a kind of critical assistant immune cells in solid tumor therapy to recruit cytotoxic lymphocytes and modulate tumor microenvironment. Despite continuous efforts in technological optimization, increasing research, documentation, and data transparency is also critical for the clinical success of PSC-based immunotherapy. There is definitely a demand for further peer-reviewed clinical trial outcomes involving PSC-derived immune cells, which will facilitate this technology to become a real life-saving therapy.
